# Arbuscular Mycorrhiza Mediates Efficient Recycling From Soil to Plants of Nitrogen Bound in Chitin

**DOI:** 10.3389/fmicb.2021.574060

**Published:** 2021-02-19

**Authors:** Petra Bukovská, Martin Rozmoš, Michala Kotianová, Kateřina Gančarčíková, Martin Dudáš, Hana Hršelová, Jan Jansa

**Affiliations:** Laboratory of Fungal Biology, Institute of Microbiology, Czech Academy of Sciences, Praha, Czechia

**Keywords:** chitin, microbial community, mineralization, organic nutrients, root-free zone, stable isotopic labeling, arbuscular mycorrhizal (AM) symbiosis, environmental nitrogen (N) losses

## Abstract

Symbiosis between plants and arbuscular mycorrhizal (AM) fungi, involving great majority of extant plant species including most crops, is heavily implicated in plant mineral nutrition, abiotic and biotic stress tolerance, soil aggregate stabilization, as well as shaping soil microbiomes. The latter is particularly important for efficient recycling from soil to plants of nutrients such as phosphorus and nitrogen (N) bound in organic forms. Chitin is one of the most widespread polysaccharides on Earth, and contains substantial amounts of N (>6% by weight). Chitin is present in insect exoskeletons and cell walls of many fungi, and can be degraded by many prokaryotic as well as eukaryotic microbes normally present in soil. However, the AM fungi seem not to have the ability to directly access N bound in chitin molecules, thus relying on microbes in their hyphosphere to gain access to this nutrient-rich resource in the process referred to as organic N mineralization. Here we show, using data from two pot experiments, both including root-free compartments amended with ^15^N-labeled chitin, that AM fungi can channel substantial proportions (more than 20%) of N supplied as chitin into their plants hosts within as short as 5 weeks. Further, we show that overall N losses (leaching and/or volatilization), sometimes exceeding 50% of the N supplied to the soil as chitin within several weeks, were significantly lower in mycorrhizal as compared to non-mycorrhizal pots. Surprisingly, the rate of chitin mineralization and its N utilization by the AM fungi was at least as fast as that of green manure (clover biomass), based on direct ^15^N labeling and tracing. This efficient N recycling from soil to plant, observed in mycorrhizal pots, was not strongly affected by the composition of AM fungal communities or environmental context (glasshouse or outdoors, additional mineral N supply to the plants or not). These results indicate that AM fungi in general can be regarded as a critical and robust soil resource with respect to complex soil processes such as organic N mineralization and recycling. More specific research is warranted into the exact molecular mechanisms and microbial players behind the observed patterns.

## Introduction

Arbuscular mycorrhizal (AM) symbiosis is widespread and often mutually beneficial mode of coexistence of plants with certain fungi, important for functioning of terrestrial ecosystems and food and feed production for humans (Rillig, 2004; Smith and Read, 2008; Brundrett and Tedersoo, 2018). It involves majority of extant plant species and specialized soil fungi from Glomeromycotina and Mucoromycotina (Field and Pressel, 2018). It is considered evolutionarily ancient/primordial as compared to other kinds of mycorrhizal symbiosis as well as to rhizobial symbioses involved in biological dinitrogen fixation for their leguminous host plants (Parniske, 2008).

The AM fungi colonize both roots and soil and literally interconnect those two environments, playing particularly important roles in acquisition by plants of nutrients with limited mobility in soil such as phosphorus (P) and zinc (Jansa et al., 2003; Smith et al., 2004). The AM symbiosis also significantly affects composition and functioning of soil microbiome (de Boer et al., 2005; Artursson et al., 2006; Hartmann et al., 2009), multitrophic interactions involving plant aboveground parts (Babikova et al., 2013; Duhamel et al., 2013; Charters et al., 2020), and also physicochemical properties of soil such as aggregate stability (Rillig and Mummey, 2006). Due to generally low specificity of the partner choice in AM symbiosis, different plant individuals, belonging to the same or different plant species, can be interconnected by a shared AM fungal mycelium, forming so called common mycorrhizal networks (Kennedy et al., 2003; Simard and Durall, 2004). These structures can have far-reaching consequences for redistribution of symbiotic benefits and costs within plant communities (Walder et al., 2012; Fellbaum et al., 2014) and eventually affect vegetation structure of the ecosystems (Bever et al., 2010). This symbiosis has also previously been demonstrated to affect drought resistance of its plant host (Augé et al., 2015), most likely indirectly, not via significant water uptake through the AM fungal hyphae (George et al., 1992; Püschel et al., 2020).

The evidence for AM symbiosis being involved in nitrogen (N) nutrition of the host plants and soil N cycling is less elaborated/more equivocal than that for P cycling (Smith and Read, 2008; Jansa et al., 2011; Bücking and Kafle, 2015). Yet, it seems that the symbiosis may indeed significantly and directly contribute to plant N uptake from diffusion-limited sources such as soil NH_4_^+^ pool, particularly in alkaline soils. Further, the AM fungi obviously affect mineralization of organic N in soil and increase N uptake by the plants from decomposing organic materials (Johansen et al., 1992, 1993; Hodge et al., 2001; Jansa et al., 2019; Thirkell et al., 2019). Lowering gaseous (including N_2_O emissions) and liquid (leaching) losses from soil due to AM symbiosis establishment were also previously reported (Bender et al., 2015; Cavagnaro et al., 2015; Bowles et al., 2018; Storer et al., 2018). However, not always has such efficient AM symbiosis-mediated uptake of N been observed from organic materials to plants (Hodge et al., 2000; Hodge, 2001). This lack of consistent evidence for efficient N supply to plants from organic N sources in soil may be due to different aspects of the experimental systems (such as soil/substrate properties, microbial inputs, identity of AM fungi, and/or N forms) employed in different studies (Bender et al., 2015; Hodge and Storer, 2015; Kohl and van der Heijden, 2016; Paymaneh et al., 2018). One important determinant seems the variable demand of plant for the N, i.e., resource stoichiometry context, where plant growth could be limited by N or by another resource (Johnson, 2010; Johnson et al., 2015). Another important aspect seems to be the intrinsic requirements of the AM fungi and/or other soil microbes for N. This may lead to competition for free mineral N in soil solution between the plant and the microbes including the AM fungi, particularly at low N availabilities (Hodge et al., 2010; Püschel et al., 2016).

To improve our understanding of context-dependency of the effects of AM symbiosis on organic N recycling in soil-plant systems, we conducted two pot experiments, where ^15^N-labeled organic materials (either chitin or clover biomass) were supplied patchily to AM fungal hyphosphere (i.e., they only were directly accessible to AM fungal hyphae and not to roots). In those experiments, we quantified transfer of ^15^N to the plants and also to other system compartments, and the retention of ^15^N in the labeling zones. These analyses allowed assembling complete ^15^N budgets on a per-pot basis (where losses, either gaseous or liquid, were quantified by subtraction of excess ^15^N measured at the end of the experiment in all available system compartments from the excess ^15^N supplied to the pots upon their establishment). Here we tested two different N supply levels to plants, different AM fungal communities, and environmental (microclimatic) conditions that potentially all could have affected the soil N cycling. Along with nutrient/isotopic analyses of the plant/potting substrate samples, we also characterized development of specific microbial guilds (including nitrification bacteria) potentially relevant for soil N cycling in the different system compartments and throughout time. Experiment 2 has previously been described in context of an independent study (Bukovská et al., 2018). There is very little overlap of data presented in that previous publication and in this manuscript, except a part of ^15^N transfer data to plants inoculated with *Rhizophagus* in Experiment 2. The novelty of this current manuscript is in presenting the entire ^15^N budgets on a per-pot basis in both of the experiments described here, additional data from Experiment 2 (e.g., comparison of glasshouse with outdoor conditions, and inclusion of multispecies AM fungal inoculants) and particularly the temporal dynamics in Experiment 1.

## Materials and Methods

### Experimental Containers and Potting Substrate

**FIGURE 1:**
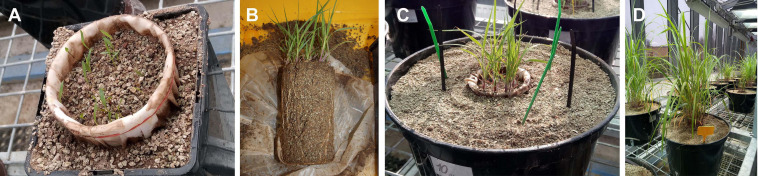
Plant root compartments for Experiment 1 lined with root-barrier mesh and placed in 2 L pots at 1 week after seeding **(A)** and the root development of the plants at 4 weeks after the seeding **(B)**. Experimental containers of Experiment 1 just after settling in the plant compartments pre-grown for 4 weeks, and the different (organic and mineral) nitrogen traps in 10 L pots **(C)**, and after further 8 weeks of growth just before the destructive harvest **(D)**.

Both of the experiments described here were carried out in 10 L pots filled with a potting substrate composed of 10% field soil from Litoměřice, Czech Republic (Řezáčová et al., 2016), sterilized by γ-rays (>25 kGy), 45% zeolite, grain size <2.5 mm (Zeopol, Břeclav sro, Czech Republic), autoclaved at 121°C for 1 h, and 45% sand (autoclaved at 121°C for 1 h), mixed by volume. This substrate had been used in several previous experiments (Bukovská et al., 2016; Püschel et al., 2016, 2020; Řezáčová et al., 2018; Jansa et al., 2020) and had the following properties: pH (water) = 8.9, total P (incineration at 550°C and acid digestion) = 46.5 mg kg^–1^, water extractable P = 2.95 mg kg^–1^, total N = 0.0132%, total organic C = 0.222%. Plant root growth was confined in both of the experiments described here to 500 ml volume delimited by plastic containers as in Figure 1A (cheese forms P00718, Anelli SRL, Montanaso, Italy), lined with 42 μm nylon mesh shown in Figure 1B (Uhelon 130T, Silk and Progress, Brněnec, Czech Republic).

### Biological Inputs—Plants and Microbes

Plant containers were the only recipients of AM fungal inoculum, which was either open-pot produced with leek (*Allium porrum*) as a host plant, or produced in monoxenic *in vitro* systems together with cichory (*Cichorium intybus*) Ri-T DNA transformed root organ cultures. A mixture of soil microbes (contained in potting substrate of leek (*Allium porrum*) cultures devoid of AM fungi, grown in a glasshouse for more than 2 years, also called non-mycorrhizal or mock inoculum) were applied into all system compartments. For more details of the inoculum production and microbial community profiling please see Gryndler et al. (2018). Both the mycorrhizal and mock inocula produced in open pots were using the same potting substrate as that used in the pot experiments described here. Leek roots from the mock inoculum cultures were chopped to fragments <5 mm and mixed in the potting substrate along with the mock inoculum. In Experiment 1, 5% of the substrate volume of the mock inoculum were applied throughout, whereas 1% of the mock inoculum into the potting substrate were applied in Experiment 2.

### Timeline, Environmental Conditions, Watering, and Fertilization of the Plants

Plant compartments were initially established in 2 L pots (Figure 1A) and sown with approximately 50 seeds of *Andropogon gerardii* (Jelitto Staudensamen, Schwarmstedt, Germany), 4 weeks prior to setting up the large experimental pots (Figure 1C). The seedlings were not fertilized at this stage at all, only deionized water was provided so as to maintain the water holding capacity of the substrate at around 80%. Thereafter, plant compartments with pre-grown plants/AM fungi were transferred into large (10 L) pots to facilitate rapid development of AM hyphal networks throughout the pots. Plants were pre-grown in the small (2 L) pots in the glasshouse, whereas the large (10 L) pots were kept either in the glasshouse or outdoors (the latter applicable to part of the Experiment 2 only). Plants in the glasshouse were provided with supplemental light (extending the photoperiod to 14 h), with a minimum intensity of 200 μmol photosynthetically active radiation m^–2^ s^–1^ throughout the photoperiod. The plants (i.e., the plant compartments) were fertilized on a weekly basis starting from week 4 after transfer to the large pots with 60 ml Long-Ashton nutrient solution (Hewitt, 1966) with the P concentration reduced to 20% of the original recipe and with ambient or threefold (3 × N) higher concentration of N as in the original recipe (in Experiment 1) or from week 1 after transfer to the large pots with ambient Long Ashton nutrient solution in Experiment 2 as previously (Bukovská et al., 2018). Each plant compartment thus received 2.4 or 7.2 mmol N in the ambient or 3 × N regime, respectively, and 0.078 mmol P with the nutrient solution throughout plant growth in any of the experiments. The pots were watered daily with deionized water to maintain approximately 80% of the water holding capacity of the substrate (to prevent leaching).

### Organic N Supply Into the Root-Free Zone—Trap Application and Recovery

In the root-free zone beyond the reach of plant roots, at a distance of 3 cm from the plant compartment and about 5 cm below substrate surface, six or eight AM hyphal trap compartments were embedded in Experiments 1 or 2, respectively. These hyphal traps (cylinders with 3.5 cm inner diameter and length of 3 cm, opening toward the plant compartment) were filled with the same potting substrate (45 ml each) as the rest of the experimental pots and covered at both openings with 206 μm mesh (Uhelon 35S, Silk and Progress). The traps in Experiment 1 were added with zygomycetous cell walls composed nearly exclusively of chitin (Jansa et al., 2020) at a rate of 0.78 mmol N/trap (and containing also 96.3 μmol P/trap) or the corresponding amounts of N and P in mineral forms (sodium nitrate or sodium phosphate, respectively). Three chitin-traps and three mineral NP control traps were added into each pot in Experiment 1 (Figure 1C). The details of the amendments used in Experiment 2 have already been described elsewhere (Bukovská et al., 2018)—briefly, there was always the same suite of traps added into each pot, one of eight was amended with zygomycetous cell walls and one with clover biomass. Only one of the traps in each pot (either chitin or clover) was labeled with ^15^N, depending on the treatment (see below). The traps were harvested at 3, 5, or 8 weeks after planting in Experiment 1 and at a single timepoint (5 weeks after planting) in Experiment 2. Chitin labeled or not with ^15^N (>99 atom% ^15^N, Experiment 1) or dually with both ^15^N and ^13^C (98 atom% ^15^N and 49 atom% ^13^C, Experiment 2) was produced by growing *Zygorrhynchus* sp. on mineral media supplemented with ^15^N-ammonium sulfate or ^15^N-ammonium sulfate and ^13^C-glucose as described previously (Bukovská et al., 2018). ^15^N-labeled clover biomass (50 atom% ^15^N) was used in Experiment 2 (in a different set of pots from the labeled chitin) and details pertinent to its preparation and dosage have been published previously (Bukovská et al., 2018).

### Factorial Structure of Experiments 1 and 2, and Mycorrhizal Inoculation Treatments

In Experiment 1, five mycorrhizal pots (inoculated with 24,000 *in vitro* produced spores of *Rhizophagus irregularis* SYM5 genotype with accompanying hyphae applied per each plant compartment before seeding) and five non-mycorrhizal control pots were included in each of the ambient and 3 × N regimes, totaling 20 pots, where ^15^N-labeled chitin was applied in the corresponding hyphal traps. Additionally, 4 mycorrhizal and 4 non-mycorrhizal pots were established, where chitin with natural ^15^N abundance was used (isotopic controls). Climatic data during the plant growth are provided in the electronic supplements to this paper (Supplementary Table 1).

In Experiment 2: sixteen pots with ^15^N-labeled chitin were included in the design, eight of which were added with pot-produced *Rhizophagus irregularis* BEG 158 inoculum (50 ml applied per plant compartment), and eight remained non-mycorrhizal. Four mycorrhizal and four non-mycorrhizal pots were placed in the glasshouse and four pots of each of the inoculation treatments were kept outdoors—climatic data for both environments are provided among supplementary data to this paper (Supplementary Table 2).

Moreover, additional sixteen pots were included in the design of Experiment 2, where ^15^N-labeled clover biomass was used in the relevant AM hyphal traps. Four such pots were inoculated with *R. irregularis* as above (further referred to as “RI” treatment). Four additional pots were inoculated with 50 ml per plant compartment of a mixture (3:1:6, by volume) of pot-produced inocula of *R. irregularis* BEG 158, *Funneliformis mosseae* BEG 161, and *Claroideoglomus claroideum* BEG 155, this treatment is further referred to as “Mix” treatment. Inoculum proportions for this treatment were tuned up so as to prevent competitive exclusion of any of the fungi and were estimated using inoculation pre-experiment described in the Supplementary Data Sheet 1. Four more pots with ^15^N-labeled clover biomass were inoculated with 50 ml of unsterile field soil from Litoměřice, Czech Republic, freshly (1 week prior to pot establishment) collected from the same site as the soil for creating potting substrate. This inoculation treatment is further referred to as “LT.” Four additional pots with ^15^N-labeled clover biomass did not receive any AM fungal inoculum (i.e., served as non-mycorrhizal controls, further referred to as “NM” treatment), but received 50 ml of the mock inoculum instead.

### Plant and Substrate Sample Collection and Processing

In Experiment 1, one pair of chitin-amended and mineral NP traps were harvested from each pot at each harvest time (i.e., at 3, 5, and 8 weeks after pot establishment). The substrate collected from the traps was subsequently dried at 65°C for 3 days and pulverized using a ball mill MM200 (Retsch, Haan, Germany) at 25 Hz for 2 min. Furthermore, three leaf tips (2-3 cm long) of *Andropogon* were clipped from each pot on a weekly basis to analyze isotopic composition of N in the leaves. Upon final harvest, 8 weeks after transferring the plant compartments into the large pots (Figure 1D), shoots and roots of the plants were collected, dried at 65°C for 3 days and pulverized using the ball mill as above. Substrate from the plant compartment and from the root-free zone adjacent to the plant compartment and surrounding the different hyphal traps was collected, dried and pulverized as above before any subsequent analyses.

### Elemental and Isotopic Analyses

Total N and C concentrations and ^15^N/^14^N and ^13^C/^12^C isotopic ratios in all plant and substrate samples were measured using elemental analyzer Flash EA2000 coupled to Delta V mass spectrometer (operating either in natural abundance or heavy enrichment modes, depending on the expected isotopic enrichments) via Conflow IV interface, using sample weights between 2 mg (plants) and 25 mg (substrates without organic amendments). Results were reported either in standard delta notation relative to Vienna Pee-Dee Belemnite standard or as atom% of the heavier isotope relative to isotopically un-labeled controls. The N concentrations and ^15^N enrichments of the individual samples were used to calculate N content of the individual system compartments and excess ^15^N budget on a per-pot basis.

The P concentrations in plant biomass samples (measured separately for shoots and roots) were assessed using dry incineration of 100 mg sample aliquots at 550°C and hot HNO_3_ extraction and subsequent colorimetry with Malachite green as described previously (Püschel et al., 2017). The P content of the plants was calculated from the P concentrations and plant dry biomass data on a per-pot basis.

### Molecular Quantification of Microbial Guilds and AM Fungi Amplicon Sequencing

Mycorrhizal colonization in both roots and substrate samples was measured with quantitative real-time PCR (qPCR) either using NS31 and AML2 primers or with genotype-specific primers and hydrolysis probes (i.e., using markers mt5, intra, moss, or clar) as specified in the Supplementary Table 3. Furthermore, abundance of eubacteria, bacterial ammonia oxidizers, fungi, and protists was quantified in substrate samples from Experiment 1 as specified in the Molecular toolbox provided as a Supplementary Table 3 to this paper. All qPCR data were corrected for the recovery of the internal DNA standard spiked to each sample before DNA extraction and assessed with a specific molecular marker described previously (Thonar et al., 2012) and detailed also in the Molecular toolbox in the Supplementary Table 3.

Composition of AM fungal communities in the plant and substrate samples of the LT treatment (and roots from the RI and Mix treatment used as a reference) was analyzed using dually indexed (Nextera XT) amplicons generated with WANDA and AML2 primers (see Molecular toolbox in the Supplementary Table 3) on Illumina MiSeq 2 × 300 platform. Processing of the sequencing output was carried out in Seed software (Vĕtrovský and Baldrian, 2013) and external software packages detailed elsewhere (Jansa et al., 2020). Briefly, raw sequence reads were paired with a minimum of 40 bp overlap, quality filtered (only sequences with quality score ≥30 were retained), primers (no mismatches allowed) were cut off the sequences, the sequences filtered out to remove possible chimeras, clustered at 97% similarity threshold to operational taxonomic units (OTUs) and the OTUs then identified (blasted) using the SILVA database. This allowed identification of Glomeromycotan vs. other sequences, of which the latter were all removed from the dataset at this stage. Thereafter, sequencing depth of individual samples were rarefied to 2,000 reads per sample, clustered at 97% similarity threshold and identified by comparing most abundant sequence from each cluster with in-house customized sequence reference database (based mainly on full length Krüger fragment sequencing) at *E*-value limit <10^–150^. Thereafter, singletons (i.e., OTU composed of just a single sequence) were removed, relative abundances of OTUs per sample calculated, and individual OTUs identified as the same AM fungal genus pooled together.

### Statistics

Statistical evaluation of data was carried out in Statgraphics Plus for Windows v. 3.1., using the following tools: Descriptive statistics (calculation of means and standard errors of means to draw figures), one- or multifactor analyses of variance (ANOVA) or analyses of covariance (ANCOVA), general linear models (GLM), followed by Tukey HSD *post hoc* test for mean separation in case where significant effects of experimental factors were detected. Data were always carefully checked so as not to violate assumptions of the respective statistical tests—in case of significant violation of test assumptions such as significant data heteroscedasticity, corrective data transformation or unparametric alternatives were sought. All raw data used for statistical analyses and assembling illustrations (which was done in SigmaPlot for Windows v. 13.0) are provided in Supplementary Table 4.

## Results

### Experiment 1: ^15^N Isotopic Tracing

**FIGURE 2:**
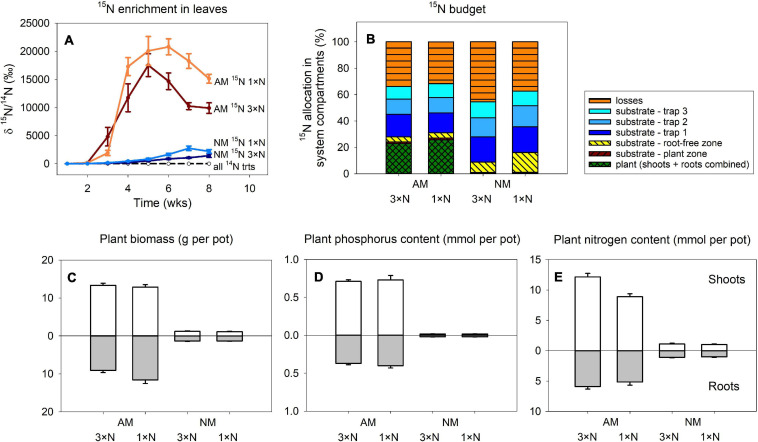
Time-course of ^15^N enrichment in plant leaves **(A)** and the ^15^N budget (i.e., allocation of excess ^15^N supplied to the pots with isotopically labeled chitin) in Experiment 1 **(B)** as affected by presence of the arbuscular mycorrhizal (AM) fungus as compared to non-mycorrhizal (NM) control treatment, where plants were either supplied with surplus nitrogen (3 × N) or grown under ambient conditions (1 × N). Traps 1 through 3 in **(B)** refer to ^15^N-labeled chitin traps harvested at 3, 5, and 8 weeks after setting up the pots, respectively. Plant biomass production **(C)**, phosphorus content **(D)** and nitrogen content **(E)** in the shoots and roots are shown for the different treatment combinations. Treatment means (*n* = 5) are shown except the ^14^N treatments in **(A)**, where *n* = 8. Error bars (where applicable) indicate ±1 **(A)** or +1 **(C–E)** standard error of the mean.

The ^15^N isotopic enrichment of leaf tips sampled throughout the Experiment 1 (Figure 2A, only data from 20 pots amended with ^15^N-labeled chitin included into GLM analysis presented here) was significantly affected by both categorical factors, i.e., presence of AM fungus [*F*_(__1_,_155__)_ = 154, *p* < 0.001] and N fertilization of the plant [*F*_(__1_,_155__)_ = 5.53, *p* = 0.02], as well as by sampling time (quantitative factor): *F*_(__1_,_155__)_ = 69.6, *p* < 0.001. Interaction between the two categorical factors was not significant [*F*_(__1_,_155__)_ = 2.93, p = 0.09].

The only influential factor significantly affecting the amounts of excess ^15^N allocated to the different compartments of the pots in Experiment 1 (Figure 2B), as assessed by 2-way ANOVA, was the presence of AM fungus. This factor turned to be significant (*p* < 0.05) for all system compartments except ^15^N-chitin amended traps collected at final harvest, 8 weeks after assembling the large experimental pots, for which presence of AM fungus was only marginally significant (*p* = 0.07). Generally, higher amounts of ^15^N from the chitin were allocated to plants or substrate in the plant compartment in AM fungus-inoculated treatment, whereas higher ^15^N allocation was observed in non-mycorrhizal pots in all other compartments (including the calculated ^15^N losses). The contribution of N fertilization regime of the plants or the interaction of the two main factors (i.e., mycorrhizal inoculation and the N supply levels) turned to be insignificant for ^15^N allocation to any of the system compartments (*p* > 0.05).

### Experiment 1: Plant Biomass and Mineral Nutrition

Plant variables in Experiment 1 (biomass, P and N contents) were all significantly affected by AM fungal inoculation (Figures 2C–E), with mycorrhizal plants always showing higher values than the NM plants (*p* < 0.05). Roots of mycorrhizal plants supplied with ambient N levels grew larger than if the plants received higher N concentrations in the nutrient solution (Figure 2C), resulting in significant interaction between the main factors for root biomass [*F*_(__1_,_16__)_ = 4.99, *p* = 0.04], though not for total plant biomass. For plant N content, shoots of plants receiving higher N concentration in the nutrient solution showed higher accumulation of N in shoots, resulting in significant interaction between the main factors [*F*_(__1_,_16__)_ = 17.5, *p* < 0.001], which also translated to total plant N content [where the interaction of the two main factors still held significant [*F*_(__1_,_16__)_ = 12.5, *p* = 0.003].

### Experiment 1: Development of the AM Fungus

**FIGURE 3:**
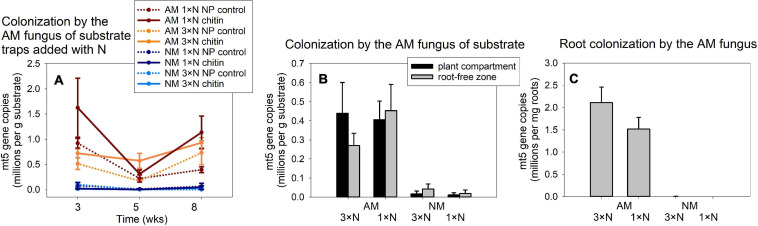
Colonization of substrate samples **(A,B)** and roots **(C)** of experimental *Andropogon gerardii* plants by the arbuscular mycorrhizal (AM) fungus *Rhizophagus irregularis* in Experiment 1, as affected by the harvest time **(A)** and plant nitrogen supply (1 × N, ambient; 3 × N, surplus nitrogen applied to the plant compartment), in the AM fungus-inoculated pots as compared with the non-mycorrhizal (NM) control treatment. Colonization was estimated by quantitative real-time PCR with specific primers and a hydrolysis probe (mt5) specifically targeting the mitochondrial large ribosomal subunit of the *R. irregularis*, and the data were corrected for internal DNA standard recovery upon DNA extraction from the different samples. The data refer to substrate traps added with nitrogen either in organic (chitin) or inorganic forms (NP control), and harvested at three different time points **(A)**, or to substrate samples recovered from plant compartment and from root-free zone adjacent to the plant compartment **(B)** and to the roots harvested at the end of the experiment at 8 weeks from setting up the large experimental pots **(C)**. Treatment means (*n* = 5) are shown, error bars indicate ±1 **(A)** or +1 **(B,C)** standard error of the mean.

The qPCR signal indicative of the development of AM fungus in Experiment 1 was strongly affected by AM fungal inoculation in all samples, where it was determined, as per three- or two-way ANOVAs for traps and the other samples, respectively (Figure 3). Because the signal in the NM samples was virtually a method background noise, we conducted further analyses (two- or one-way ANOVAs for the traps collected at different timepoints and for the other samples, respectively) only with the mycorrhizal treatment and the different timepoints in Figure 3A included as a co-variate. These analyses showed significant effect of the N form added to the traps [with chitin significantly stimulating the development of the AM fungus as compared to mineral NP amendment, *F*_(__1_,_55__)_ = 6.87, *p* = 0.011], with neither the N fertilization regime of the plants nor the harvest time having a consistent effect on the trap colonization by the AM fungus. Likewise, we did not detect any significant effect of N fertilization of the plants on AM fungal development in the other system compartments, either the substrate or the roots (Figures 3B,C, analyses not shown, raw data in Supplementary Table 4).

### Experiment 1: Rhizosphere and Hyphosphere Microbiome Analyses

**FIGURE 4:**
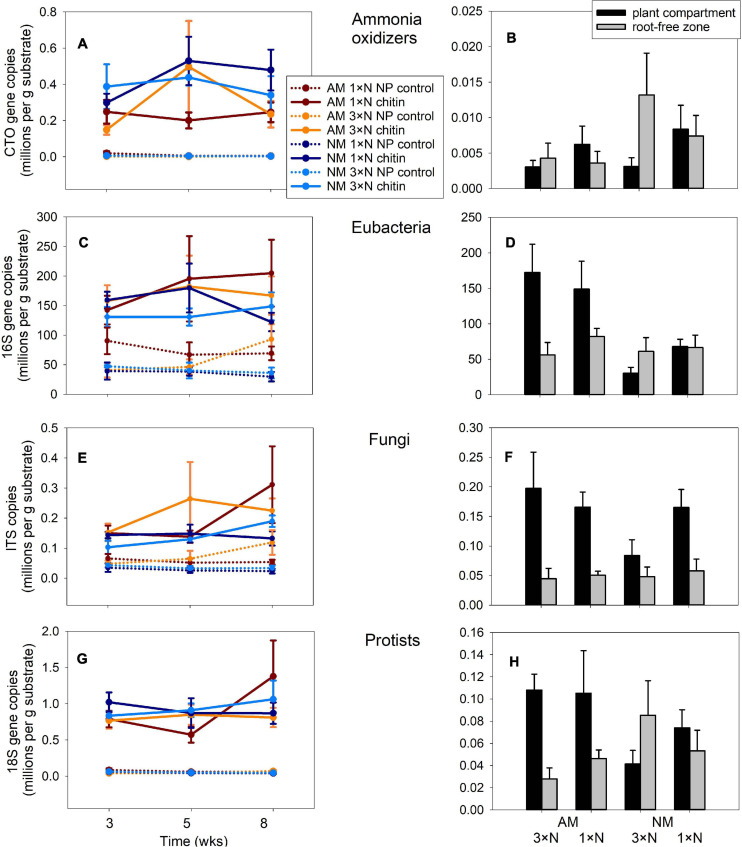
Quantification of different microbial guilds in the substrate samples recovered from Experiment 1 by quantitative real-time PCR with group-specific primers, targeting 16S of ammonia oxidizing bacteria **(A,B)**, or eubacteria **(C,D)**, internal transcribed spacer 1 of fungi **(E,F)** or 18S of protists **(G,H)**. The data refer to substrate traps added with nitrogen either in organic (chitin) or inorganic forms (NP control), and harvested at three different time points **(A,C,E,G)**, or to substrate samples recovered from plant compartment and from root-free zone adjacent to the plant compartment **(B,D,F,H)** harvested at the end of the experiment at 8 weeks from setting up the large experimental pots. Treatment means (*n* = 5) are shown, error bars indicate ±1 **(A,C,E,G)** or +1 **(B,D,F,H)** standard error of the mean.

Three-way ANCOVA, where harvest time of the traps was used as a covariate, indicated strong effect of N form (organic vs. mineral) added to the AM hyphal traps with respect to the community size of all four assessed microbial guilds (*p* < 0.001 in all cases). Chitin stimulated higher abundances of ammonia oxidizers (Figure 4A), bacteria (Figure 4C), fungi (Figure 4E) as well as protists (Figure 4G) as compared to the NP controls. Presence of the AM fungus in the hyphal traps had variable effects on different microbial guilds, with ammonia oxidizers being significantly suppressed by the AM fungus [*F*_(__1_,_111__)_ = 5.50, *p* = 0.021], eubacteria and fungi in the hyphal traps being both stimulated by presence of the AM fungus [*F*_(__1_,_111__)_ = 6.94, *p* = 0.01 and *F*_(__1_,_111__)_ = 8.92, *p* = 0.004, respectively] and protists being not affected (*p* > 0.05). Harvest time only had a significant effect on abundance of fungi [*F*_(__1_,_111__)_ = 4.46, *p* = 0.037], but this turned to be only of lesser importance as compared to the effects of both main factors [i.e., N form and AM inoculation, *F*_(__1_,_111__)_ = 54.6, *p* < 0.001 and *F*_(__1_,_111__)_ = 8.92, *p* < 0.004, respectively].

In the substrate of plant compartments, abundance of ammonia oxidizers (Figure 4B) and fungi (Figure 4F) were significantly affected by neither of the experimental factors, i.e., AM fungal presence or N fertilization of the plant (analyses not shown). In contrast, AM fungal presence stimulated community size of eubacteria and protists in the plant compartment [Figures 4D,H, *F*_(__1_,_24__)_ = 17.4, *p* < 0.001 and *F*_(__1_,_24__)_ = 6.2, *p* = 0.02, respectively]. In the root-free zone adjacent to the plant compartment, only the abundance of protists was significantly affected, interactively by both AM fungal presence and N fertilization of the plants [*F*_(__1_,_24__)_ = 4.92, *p* = 0.04]. Subsequent one-way ANOVA comparisons of the effects exerted by individual factors on protist abundances turned to be all insignificant at *p* = 0.05 threshold level, however (analyses not shown).

### Experiment 2: ^15^N and ^13^C Isotopic Tracing

**FIGURE 5:**
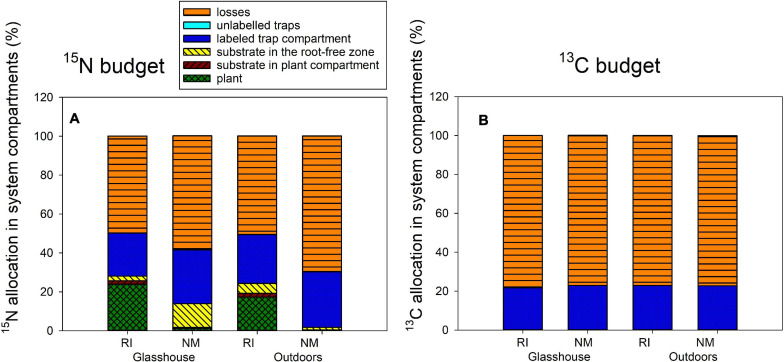
The ^15^N **(A)** and ^13^C **(B)** budgets (i.e., allocation of excess ^15^N and excess ^13^C supplied to the pots with dually labeled chitin) in the Experiment 2, as affected by presence of the arbuscular mycorrhizal fungus *Rhizophagus irregularis* (RI) as compared to the non-mycorrhizal control treatment (NM), where the pots were incubated either in the glasshouse or outdoors. Height of the stacked bars correspond to treatment means (*n* = 4).

Transfer of ^15^N to the plants from isotopically labeled chitin (Figure 5A) was strongly affected by presence of *R. irregularis* [*F*_(__1_,_12__)_ = 213, *p* < 0.001], and less strongly, but still significantly by the microclimatic conditions, i.e., glasshouse vs. outdoors [*F*_(__1_,_12__)_ = 8.0, *p* = 0.015], without significant interaction between the two factors, as revealed by two-way ANOVA. The ^15^N content in the substrate of the plant compartment was only significantly affected by presence of the AM fungus in the system, the values being significantly higher in mycorrhizal as compared to NM pots [*F*_(__1_,_12__)_ = 62.8, *p* < 0.001]. In all other (root-free) system compartments and in the calculated ^15^N losses from the system, the AM symbiosis but not microclimatic conditions strongly reduced ^15^N allocation to those compartments (*p* < 0.01), except the root-free compartment adjacent to the plant compartment. In the latter, the ^15^N allocation was affected interactively by both of the main factors [*F*_(__1_,_12__)_ = 8.75, *p* = 0.012], although subsequent one-way ANOVAs conducted for each of the environments separately did not show significant effect of AM fungus in any of the environments (analyses not shown).

The fate of ^13^C supplied with the isotopically labeled chitin was apparently not affected by any of the experimental factor in any of the significant pools (i.e., the ^13^C remainder in the chitin-amended hyphal trap and the ^13^C losses from the system, Figure 5B, analyses not shown). Yet, the statistics indicated significant difference in ^13^C allocation to unlabeled traps as affected by microclimatic conditions (see Supplementary Table 2 for exact values), but this seems to be an artifact of calculation (missing true isotopic control outdoors) rather than any important experimental findings.

**FIGURE 6:**
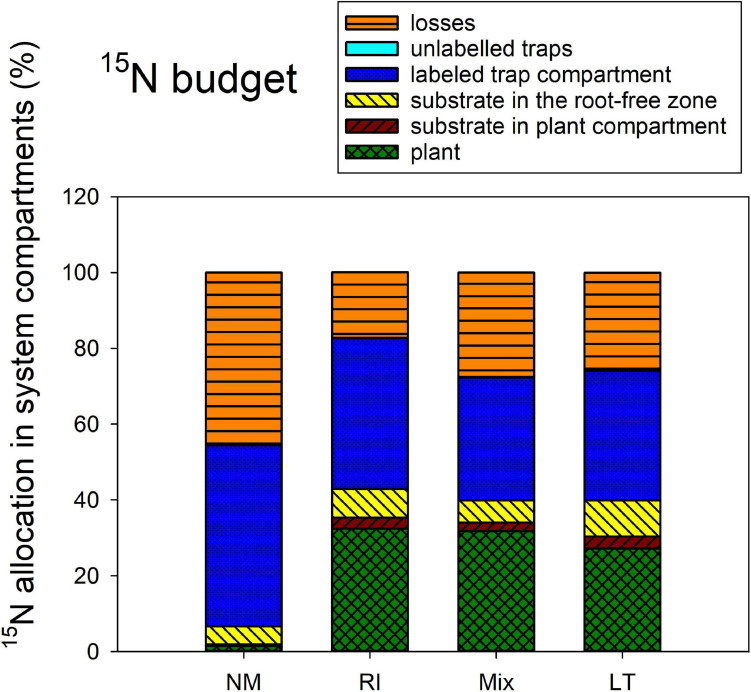
The ^15^N budget (i.e., allocation of excess ^15^N supplied to the pots with isotopically labeled clover biomass) in the Experiment 2, as affected by presence of different arbuscular mycorrhizal fungal communities, and compared to the non-mycorrhizal control treatment (NM). RI represent the treatment, where plants were inoculated with *Rhizophagus irregularis* only, Mix stands for a three-species inoculum consisting of pure strains of *R. irregularis*, *Claroideoglomus claroideum*, and *Funneliformis mosseae*, and LT stands for a treatment, where plants were inoculated by unsterile field soil from Litoměřice, Czech Republic. Height of the stacked bars correspond to treatment means (*n* = 4).

The ^15^N allocation to the different system compartments from the isotopically labeled clover biomass (Figure 6) was assessed by one-way ANOVA and subsequent Tukey HSD *post hoc* test, addressing the differences between the different AM inoculation treatments. All mycorrhizal treatments strongly differed from the NM treatment in ^15^N amount transferred to the plant [*F*_(__3_,_12__)_ = 58.5, *p* < 0.001], but there were no significant differences between the three mycorrhizal treatments themselves. There also were significant differences in ^15^N allocated to the substrate in plant compartment [*F*_(__3_,_12__)_ = 3.72, *p* = 0.042], but actually it was only the LT treatment significantly higher from the NM as per the *post hoc* test. There were no significant differences between the inoculation treatments in terms of ^15^N allocation to root-free zone adjacent to the plant compartment (analysis not shown). Yet, there was significantly [*F*_(__3_,_12__)_ = 5.38, *p* = 0.014] less ^15^N left over in the clover biomass-amended substrate trap in the LT and Mix treatments as compared to the NM control treatment. A similar pattern was observed for the ^15^N losses, which were significantly [*F*_(__3_,_12__)_ = 11.8, *p* < 0.001] higher in the NM treatment as compared to any of the mycorrhizal treatments. Allocation of ^15^N to the other (isotopically unlabeled) substrate traps was significantly [*F*_(__3_,_12__)_ = 18.0, *p* < 0.001] different between the inoculation treatments, with the LT treatment showing consistently higher values than those encountered in the NM treatment.

### Experiment 2: Development of the AM Fungi

**FIGURE 7:**
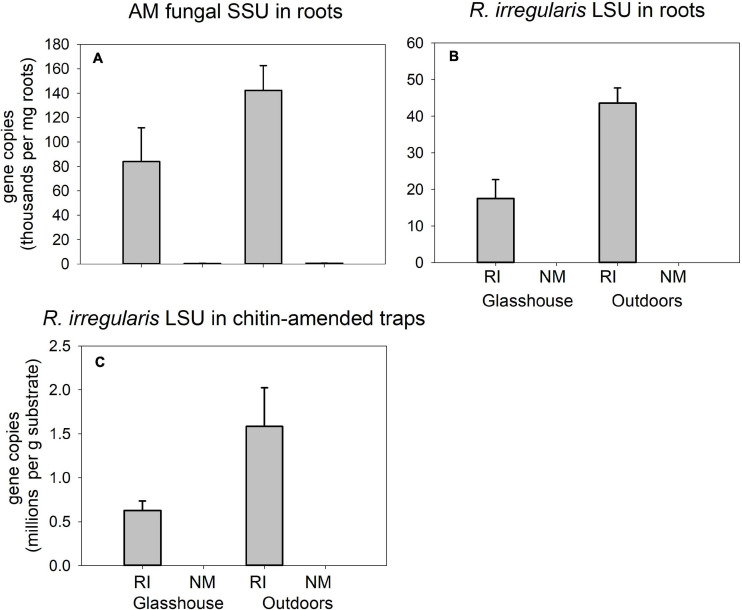
Quantification of development of *Rhizophagus irregularis* in mycorrhiza-inoculated (RI) and in the non-mycorrhizal control (NM) pots amended with isotopically labeled chitin in Experiment 2, and incubated either in the glasshouse or outdoors, as per broadly specific arbuscular mycorrhizal (AM) fungal primers NS31-AML2 targeting small ribosomal subunit (SSU) in roots **(A)** or *Rhizophagus*-specific primers with a hydrolysis probe targeting large ribosomal subunit (LSU) of *R. irregularis* in roots **(B)** and in substrate samples amended with chitin **(C)**. Results were corrected for internal DNA standard recovery upon DNA extraction from the different samples. Mean values (*n* = 4) are shown, error bars indicate +1 standard error of mean.

Strong differences were encountered between the RI-inoculated and NM pots supplied with isotopically labeled chitin (Figure 7) in the qPCR signal using both AM fungal-species unspecific (Figure 7A) and specific assays aiming at quantification of AM fungal biomass in plant roots (Figure 7B) and in the chitin-amended traps (Figure 7C) in pots (*p* < 0.001 in all three displayed cases). In case of *Rhizophagus*-specific assay targeting the DNA extracted from roots (Figure 7B), additionally, we encountered significant differences in the signal strength between the different environments, indicated by a significant interaction in two-way ANOVA analysis (not shown) and subsequent one-way ANOVA only comparing mycorrhizal samples from the glasshouse and outdoors [*F*_(__1_,_6__)_ = 15.4, *p* = 0.008].

**FIGURE 8:**
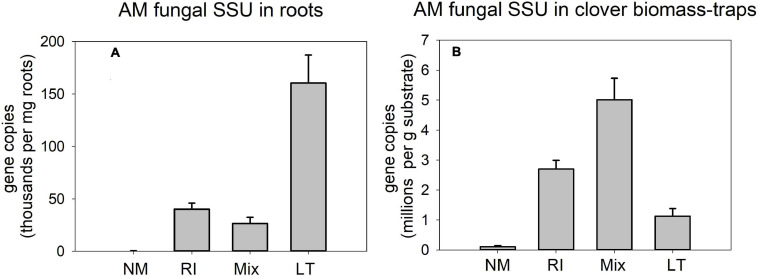
Quantification of development of arbuscular mycorrhizal fungi in the pots amended with isotopically labeled clover biomass in Experiment 2, as per broadly specific arbuscular mycorrhizal (AM) fungal primers NS31-AML2 targeting small ribosomal subunit (SSU) in roots **(A)** and in substrate samples amended with the clover biomass **(B)**. Results were corrected for internal DNA standard recovery upon DNA extraction from the different samples. RI represent the treatment, where plants were inoculated with *Rhizophagus irregularis* only, Mix stands for a three-species inoculum consisting of pure strains of *R. irregularis*, *Claroideoglomus claroideum*, and *Funneliformis mosseae*, and LT stands for a treatment, where plants were inoculated by unsterile field soil from Litoměřice, Czech Republic. Mean values (*n* = 4) are shown, error bars indicate +1 standard error of mean.

Quantification of AM fungal development in the different inoculation treatments by AM fungal-species unspecific qPCR assay in pots supplied with isotopically labeled clover biomass (Figure 8) showed strong differences between the treatments both in roots [Figure 8A, *F*_(__3_,_12__)_ = 25.8, *p* < 0.001] and clover biomass amended traps [Figure 8B, *F*_(__3_,_12__)_ = 27.3, *p* < 0.001]. However, the patterns differed between the compartments: Whereas the LT treatment showed the highest values in roots as compared to all other treatments, the highest signal in the traps was encountered for the Mix treatment, whereas the signal in the clover biomass-amended traps recovered from the LT treatment was not statistically different from the NM control.

### Experiment 2: AM Fungal Community Composition

**FIGURE 9:**
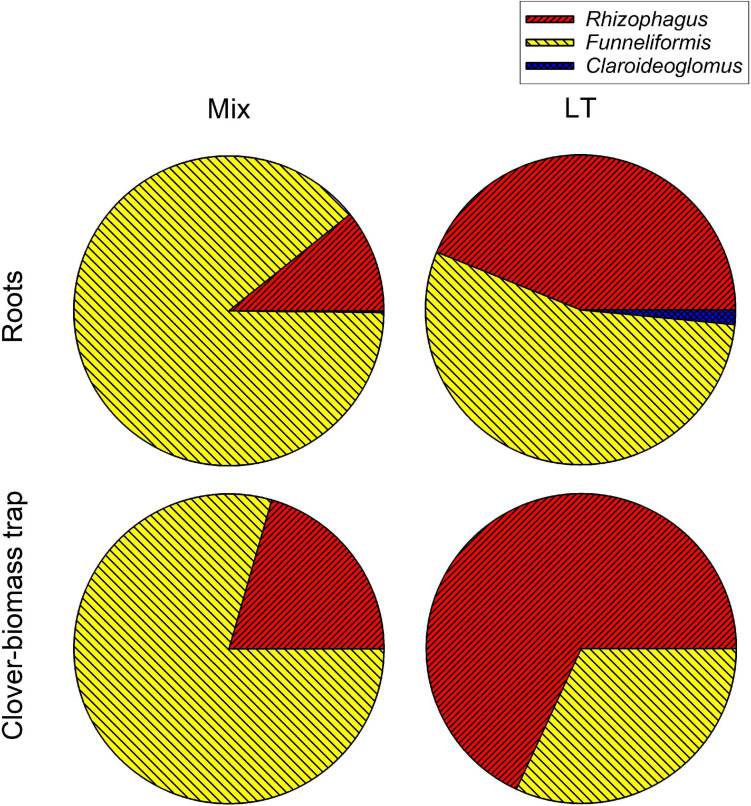
Relative composition of arbuscular mycorrhizal communities in the roots and clover-amended trap compartments in the Mix (three species) and LT (field soil) inoculation treatments in Experiment 2 as assessed by quantitative real-time PCR targeting specifically the three mycorrhizal fungal species supplied to the pots in the Mix treatment. Fractional values are means of four biological replicates (pots).

**FIGURE 10:**
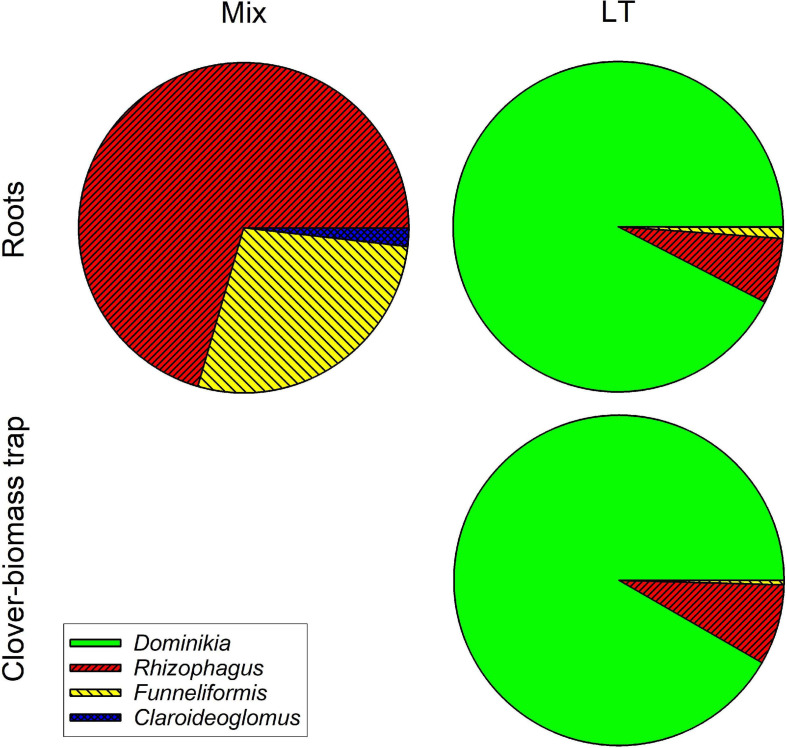
Relative composition of arbuscular mycorrhizal communities in the roots and clover-amended trap compartments in the Mix (three species) and LT (field soil) inoculation treatments in Experiment 2 as assessed by WANDA-AML2 amplicon sequencing using Illumina Miseq platform. Clusters (97% similarity) were identified using in-house customized sequence reference database and their relative abundances summed per mycorrhizal fungal genus. Fractional values are means of four biological replicates (pots).

Analysis of AM fungal community composition in the roots and clover biomass-amended traps in the Mix treatment by AM fungal species-specific qPCR assays showed predominance of *Funneliformis mosseae* in both roots and the traps, followed by *Rhizophagus irregularis* and a small fraction of *Claroideoglomus claroideum* (Figure 9). Analysis of composition of PCR amplicons generated from roots of the Mix treatment by broadly AM fungal specific primers showed more equitably balanced AM fungal community dominated by *Rhizophagus irregularis* (Figure 10). The qPCR assays using samples from the LT treatment returned mostly *Rhizophagus* and *Funneliformis* (Figure 9), but the absolute copy numbers were low (see Supplementary Table 4 for data). The analysis of composition of PCR amplicons by Illumina sequencing (Figure 10) confirmed the presence of both *Rhizophagus* and *Funneliformis* in the roots and substrate samples from the LT treatment in similar proportions as detected by qPCR, but, additionally, also a high abundance of *Dominikia* sp. (Figure 10).

## Discussion

In our current research, we observed consistent and strong facilitation of plant N uptake from the organic N source supplied into a spatially discrete patch without direct contact to the plant compartment, through the AM fungal networks, with concomitant significant reduction of N losses from the organic N source to the environment in mycorrhizal scenario (Figures 2B, 5A, 6). A large share (usually 20 or more%) of the ^15^N supplied with the organic sources was mineralized and transported to the mycorrhizal plants within as few as 5–8 weeks of incubation. The N losses in mycorrhizal pots usually reduced by as much as 10–20% of the supplied N within the same timeframe. These results do not seem to be fully explainable by the N being transported passively via mass flow through the potting substrate toward the plants. Mass flow of the solutes is likely different (i.e., faster in mycorrhizal as compared to non-mycorrhizal pots) due to the differences in size (Smith and Sperry, 2014) between mycorrhizal and non-mycorrhizal plants. Actually, the mycorrhizal effect size of ^15^N uptake was several fold stronger as compared to the mycorrhizal effects on plant biomass—see Figure 2 and also Bukovská et al. (2018) for biomass data from Experiment 2 and for more discussion. Besides, we observed a significant reduction of community size of soil nitrifiers by the presence of AM fungus in our Experiment 1, and similar observation was already reported for Experiment 2 previously (Bukovská et al., 2018). This possibly means (although direct proof is still missing) that in mycorrhizal pots, the conversion of NH_4_^+^ ions released directly from the organic matter (ammonification) or from the soil microbial loop (Bukovská et al., 2018) to more mobile nitrate ions would actually be less efficient than in the non-mycorrhizal pots. Further, here we show that the rates of transfer of N from the organic source to the plant, its retention in the substrate, as well as environmental losses were only to a minor extent affected or were fully unaffected by the other experimental factors tested here, i.e., N status of the host plant, microclimatic conditions, and the AM fungal community composition. These subjects all deserve specific discussion.

To our own surprise, data from Experiment 1 clearly showed that the rates of organic N mineralization and subsequent supply to the plants via AM fungal networks were independent from the ***plant N status***. Although ^15^N concentrations in shoot biomass (Figure 2A) showed differences between ambient and elevated N supply regimes, these differences vanished when examining ^15^N budget (Figure 2B), i.e., they only represented dilution in larger total N pool. Our results thus are contrasting with the notion that the plants would finely regulate carbon supply to the AM fungi and thus the efficiency of mycorrhizal N uptake pathway according to its needs (Tian et al., 2010; Fellbaum et al., 2012, 2014; Kafle et al., 2019). Why this did not happen in our experimental system probably needs further research.

Another interesting observation was that the ***microclimatic conditions*** had actually only a very limited (albeit detectable) influence on organic N (chitin) mineralization and transport to the plant, in contrast to the large differences observed between mycorrhizal and non-mycorrhizal pots (Figure 5A). Possibly even more interestingly, we did not detect any differences in the retention of carbon originating from the dually labeled chitin in the system due to any of the experimental factors tested (Figure 5B). This may indicate complete mineralization of chitin within 5 weeks, for which time the chitin traps were deposited in the substrate of Experiment 2, and thus complete decoupling of the N and carbon cycles, without detectable effect of temperature and/or other microclimatic conditions (see Supplementary Table 2 for details). Although there were no detectable interactive effect of mycorrhiza inoculation and microclimatic conditions on the fate of N and carbon supplied with chitin into our experimental system, it seems that the development of AM symbiosis/hyphal network was somewhat slower outdoors. Further, it seems that liquid leaching (due to uncontrolled rainfalls) might have contributed to ^15^N partitioning in our experimental pots outdoors in contrast to those placed in the glasshouse, where leaching was experimentally excluded. We deduce this from the fact that (at least some) of the molecular indicators of mycorrhizal development (Figure 7) were stronger outdoors, pointing to ontogenetically younger hyphae in those pots (Jansa et al., 2008; Voříšková et al., 2017). Furthermore, indication for N leaching in the outdoors settings is due to the fact that less ^15^N was recovered from the root-free zone (where it probably moved from the labeled traps by diffusions) in the NM treatment outdoors than in the glasshouse (Figure 5A).

Another important observation was that the mycorrhizal effect on organic N (clover biomass) mineralization and use by the host plants was not strongly dependent on ***AM fungal community composition*** (Figure 6). In spite of the communities obviously being very different in terms of their composition (Figures 9, 10) and showing distinct patterns in colonization of roots and substrate (Figure 8), resembling the most contrastive life history strategies according to Hart and Reader (2002), their effects on the fate of organic N in our experimental system were surprisingly similar (Figure 6). Yet, there were some subtle differences in ^15^N partitioning between the different system compartments due to the identity of AM fungal communities. Particularly, whereas *Rhizophagus* alone and the Mix community tended to efficiently transfer the N from the clover biomass to the plant, there is evidence that the AM fungi from the unsterile LT soil tended to hoard the N in the hyphae more than the other fungi. Particularly, we observed that there was higher reallocation of ^15^N originally supplied with the clover biomass, from the labeled to unlabeled substrate traps in the LT treatment as compared to the other inoculation treatments.

Another important finding that we like to stress here was that chitin was actually decomposing very rapidly—and faster compared to clover biomass. Although the fact that chitin is not particularly recalcitrant to decomposition in soil has already been reported (Fernandez and Koide, 2012), there still is widespread perception that chitin should be counted among the recalcitrant organic compound – which obviously is not true unless complexed with tannins, for example (Adamczyk et al., 2013). The fact that the AM fungal hyphae were able to efficiently exploit this rapidly disappearing nutrient resource and reduce environmental losses thereof was likely because the plants were inoculated with the AM fungi and pre-grown for 4 weeks in the plant compartments prior to settling up the large experimental pots with chitin traps. Thus, the plants and especially the AM fungi experienced a head start when subsequently moved into the 10 L pots with the freshly established hyphal traps.

More efficient exploitation of organic N by the mycorrhizal as compared to non-mycorrhizal plants seems like a clear take-home message from our experimental work. Indeed, this may be important in terms of environmental issues such as efficient use of N fertilizers and mitigating N pollution. However, care should be exercised not to over-interpret the current results. One weakness of our experiments is the fact that mycorrhizal and non-mycorrhizal plants grew very differently (and we eluded to the possible consequences thereof above). Second weakness is application of restrictions to root growth (root barriers), which is a very artificial feature, not really having any parallel in nature. Studies in more realistic experimental settings or directly in the fields would be very valuable in this respect indeed. And so it would have been very enlightening to carefully and to a greater (taxonomic) depth analyse microbial communities in the different system compartments of our two experiments. Here we present just a teaser—showing that soil microbes are generally much more numerous in zones supplemented with organic N, in contrast to mineral (nitrate and phosphate) amendments. We also show that at least some of the microbial guilds (e.g., bacteria and fungi) could be stimulated by the presence of AM fungal hyphae, whereas ammonia oxidizers were strongly suppressed by the AM fungus (Figure 4), replicating previous findings (Bukovská et al., 2018). These results allow us to speculate that it is the rather immobile ammonium ion that is taken by the AM fungal hyphae from the soil solution, depriving the ammonia oxidizers of their only energy resource, leading to dropping their abundance in mycorrhizal treatments. The literature on the effects of AM symbiosis on nitrifier communities is particularly full of contradictions (Veresoglou et al., 2012, 2019; Bukovská et al., 2018; Wattenburger et al., 2020), yet it seems that at least part of the problem is the fact that those prokaryotes are usually very slow growers and it is difficult to establish relevant sizes of their communities in previously sterilized substrates. In our experiments, we circumvented this limitation by adding significant amounts of microbially active mock inoculum to the potting substrate before planting. But clearly, field studies would be even more appropriate in this regards and should thus be particularly paid attention to and promoted in the future.

## Data Availability Statement

Next generation sequencing data pertinent to this work were deposited in the Sequence Read Archive (SRA, https://www.ncbi.nlm.nih.gov/sra) under BioProject accession number PRJNA640488.

## Author Contributions

PB conceived and planned both pot experiments described here, and then significantly contributed to their realization, data evaluation and interpretation (particularly, the isotopic analyses, next generation sequencing and subsequent data analysis). MR helped with realization and data analyses of Experiment 1. MK conducted most of the nutrient and isotopic analyses in Experiment 1. KG processed samples from Experiment 2 for isotopic and molecular analyses including amplicon next generation sequencing. MD conducted most of the qPCR analyses and calculations. HH supported molecular analyses in both of the experiments described here. JJ contributed to planning of both of the experiments and wrote the first draft of the manuscript. All authors contributed to data interpretation and writing, read the final version of the manuscript, and agreed with submission of the manuscript in its present form.

## Conflict of Interest

The authors declare that the research was conducted in the absence of any commercial or financial relationships that could be construed as a potential conflict of interest.

## Supplementary Material

Supplementary Table 1 Environmental conditions during Experiment 1.

Supplementary Table 2 Environmental conditions during Experiment 2.

Supplementary Table 3 PCR primers and cycling conditions.

Supplementary Table 4 Data used for drawing the figures.

Supplementary Data Sheet 1 Inoculation pre-experiment to Experiment 2.
