# Supplementary material for: Arbuscular Mycorrhiza Mediates Efficient Recycling From Soil to Plants of Nitrogen Bound in Chitin
Source: Front Microbiol. 2021 Feb 19;12:574060. doi: 10.3389/fmicb.2021.574060 (PMC7933022; doi:10.3389/fmicb.2021.574060)
Supplement: Supplementary Data Sheet 1 — Inoculation pre-experiment to Experiment 2. [file Data_Sheet_1.PDF]

Supplementary Material

Inoculation pre-experiment to the Experiment 2

In order to estimate inoculum proportions allowing concurrent establishment of all three arbuscular mycorrhizal (AM) fungal species in a three-species inoculation treatment, to apply to the Mix treatment in Experiment 2, we established a small pre-experiment in 50 ml (multiplate) containers, with the same host plant as in Experiment 2 (*Andropogon gerardii*) growing in the same substrate as that used in Experiment 2, and inoculated with the same pot-produced inocula of *Claroideoglomus claroideum*, *Funneliformis mosseae*, and *Rhizophagus irregularis* as used subsequently in Experiment 2. Different ratios of the inocula were applied either at 10% inoculation rate or 30% (x3) inoculation rate, i.e. 5 ml or 15 ml of AM inoculum per 50 ml container, respectively. A non-mycorrhizal control (NM) and monospecific inoculation treatments were also included in the design.

This pre-experiment was conducted for 4 weeks, using 3 replicate containers per inoculation treatment, plant roots after harvest being dried and milled, DNA extracted and colonization by the different AM fungal species in the DNA extracts quantified using quantitative real-time PCR with species-specific primers and hydrolysis probes as described in the main article. The values were corrected for internal DNA standard recovery as described in Thonar et al (2012). Colonization rates for each of the fungus were compared across the treatments with one-way ANOVA (all significant at $p<0.001$), followed by Tukey HSD post-hoc test at $p<0.05$.

Supplementary Figure S1

Colonization rates of *Andropogon gerardii* roots by three different AM fungal species inoculated alone or in mixtures and assessed by quantitative real-time PCR with hydrolysis probes targeting each of the fungal species separately in root DNA extracts. Mean values of three biological replicates +1SE of means are shown. Different letters indicate statistical differences between means as per Tukey HSD test ($p<0.05$) following significant ANOVA

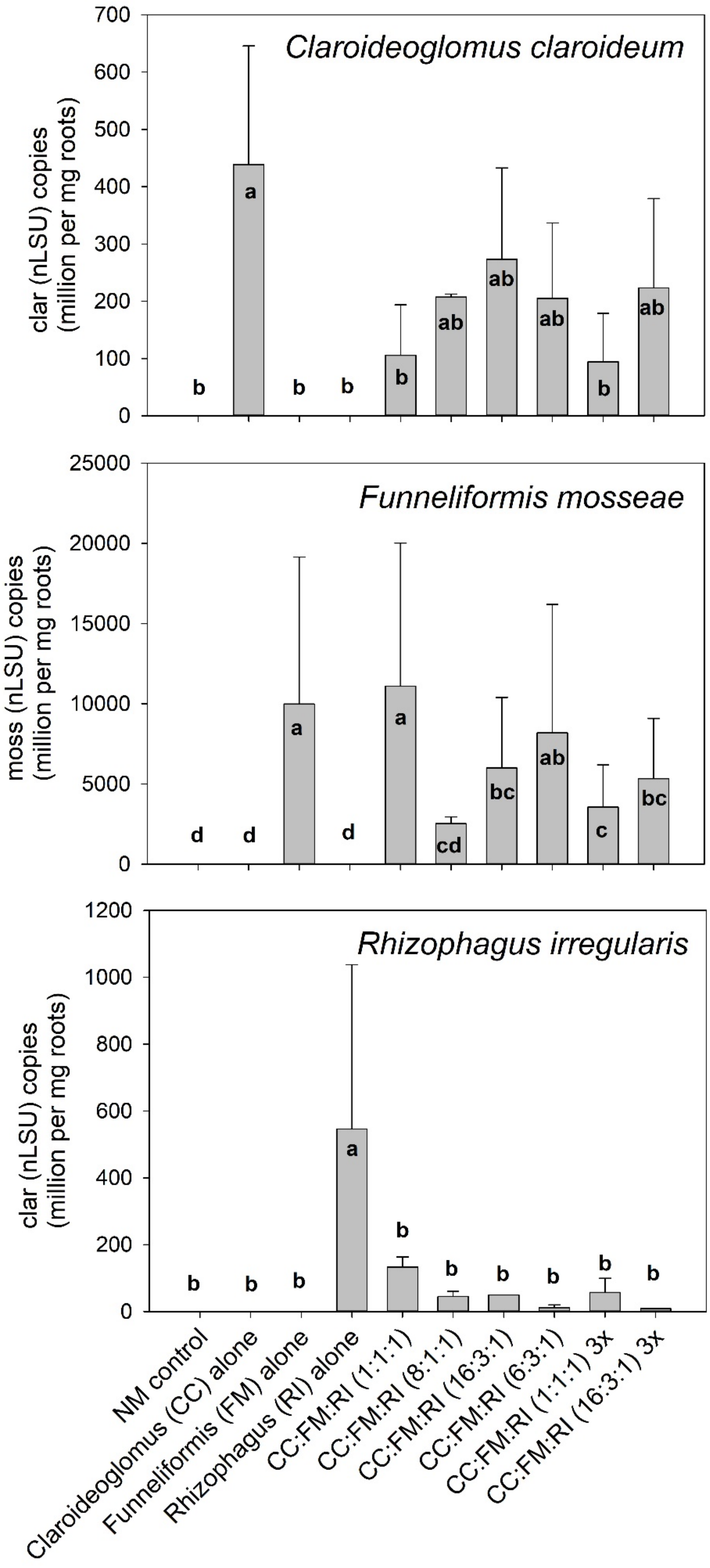
Claroideoglomus claroideum
clar (nLSU) copies (million per mg roots)
700
600
500
400
300
200
100
0
a
ab
b
Funneliformis mosseae
moss (nLSU) copies (million per mg roots)
25000
20000
15000
10000
5000
d
cd
bc
c
Rhizophagus irregularis
clar (nLSU) copies (million per mg roots)
1200
1000
800
NM control
Claroideoglomus (CC) alone
Funneliformis (FM) alone
Rhizophagus (RI) alone
CC:FM:RI (1:1:1)
CC:FM:RI (8:1:1)
CC:FM:RI (16:3:1)
CC:FM:RI (6:3:1)
CC:FM:RI (1:1:1) 3x
CC:FM:RI (16:3:1) 3x
